# Cancer associated fibroblast–derived CCL5 promotes hepatocellular carcinoma metastasis through activating HIF1α/ZEB1 axis

**DOI:** 10.1038/s41419-022-04935-1

**Published:** 2022-05-20

**Authors:** Haixu Xu, Jie Zhao, Jinping Li, Zhifeng Zhu, Zhaohai Cui, Ran Liu, Rong Lu, Zhi Yao, Qiong Xu

**Affiliations:** 1 Department of Immunology, Key Laboratory of Immune Microenvironment and Disease of the Educational Ministry of China, Tianjin Key Laboratory of Cellular and Molecular Immunology, School of Basic Medical Sciences, Tianjin Medical University, 300070 Tianjin, China; 2 Tianjin Kangzhe Pharmaceutical Technology Development Company, Ltd, 300042 Tianjin, China

**Keywords:** Stem cells, Cancer microenvironment

## Abstract

Cancer-associated fibroblasts (CAFs) are one of the most enriched components of Hepatocellular carcinoma (HCC) microenvironment, which are tightly related to the metastasis and invasion of HCC. We identified a mechanism by which CAF-derived chemokine CCL5 enhanced HCC metastasis by triggering the HIF1α/ZEB1 axis. We demonstrated that CAFs derived from HCC tissues promoted the migration and invasion of HCC cells and facilitated metastasis to the lung of NOD/SCID mice. Then the chemokine antibody array elucidated the higher chemokine CCL5 level secreted by CAFs than by paracancerous tissue fibroblasts (PTFs). Mechanistically, we found that CAF-derived CCL5 inhibited the ubiquitination and degradation of hypoxia-inducible factor 1 alpha (HIF1α) by binding to specific receptors, maintained HIF1α under normoxia, thereby up-regulated the downstream gene zinc finger enhancer-binding protein 1 (ZEB1) and induced epithelial-mesenchymal transition (EMT), ultimately validating its ability to promote lung metastasis of HCC. And this novel mechanism may have association with poor prognosis. Taken together, targeting CAF-derived CCL5 mediated HIF1α/ZEB1 cascade possibly propose a new therapeutic route for HCC.

## Introduction

Hepatocellular carcinoma (HCC) is commonly diagnosed malignancies and has a high morbidity and mortality, whose poor prognosis is mainly attributed to the occurrence of intrahepatic or systemic metastasis. The tumor microenvironment is a complex mixture, including various cytokines and stromal cells and extracellular matrix, which has important impacts on tumor outgrowth, energy metabolism, and metastasis. Cancer-associated fibroblasts (CAFs) which primarily originate in hepatic stellate cells after activation, are the most abundant components of the tumor microenvironment. Most HCC mainly develops from liver fibrosis or cirrhosis, often accompanied by activated fibroblasts accumulation. Therefore, CAFs provide a superior internal environment for the occurrence, invasion and metastasis of HCC [1].

During tumor occurrence and development, tumor cells secrete various factors to recruit CAFs into nearby. CAFs also secrete wide types of soluble factors, such as inflammatory factors, growth factors or chemokines, to reconstruct the tumor microenvironment and promote tumor cells propagation and metastasis [2–4]. It is reported that CAFs secrete CCL2, CCL5, CCL7, and CXCL16 chemokines into the tumor microenvironment, promoting the HCC cells invasion and metastasis by activating either hedgehog or TGF-β pathways [5]. Studies have indicated that CAFs upregulate the levels of CCL2, CCL26, IL-6 and LOXL2 in HCC, achieving a stronger tumor metastatic phenotype [6]. Although an accumulating number of studies have revealed the supporting function of CAFs in the form of secretory factors, the molecular regulatory mechanisms of CAFs remain incompletely defined due to the diverse biological characteristics of CAFs itself and complex tumor microenvironment components.

Hypoxia-inducible factor 1 alpha (HIF1α) promotes tumor cells to obtain stronger proliferation, invasion and metastasis capabilities under the metabolic stress conditions. Under normoxia, HIF1α is rapidly degraded by the ubiquitin-proteasome pathway, while HIF1α degradation is inhibited under hypoxic conditions. Stabilized HIF1α integrates to hypoxia response elements (HREs) and then triggers the transcription of downstream target genes that are involved in tumor angiogenesis, metastasis, and glucose metabolism [7, 8]. It has been reported that CAFs up-regulate HIF1α to strengthen the malignancy and obtain stronger invasion, metastasis and anti-chemotherapeutic abilities in prostate cancer [9]. However, studies on the correlation between CAFs and HIF1α expression in HCC cells are scarce.

Here, we demonstrated that CAFs have distinct impact on HCC metastasis. Our data indicated that CAF-derived CCL5 inhibited HIF1α ubiquitination degradation, maintained HIF1α expression under normoxia, and promoted epithelial-mesenchymal transition and lung metastasis by activating the downstream factor ZEB1. Meanwhile, CCL5 was positively correlated with HIF1α in clinical samples, these high expressions were significantly associated with poor prognosis. All the findings highlight CCL5 mediated HIF1α/ZEB1 cascade as a potential treatment strategy for preventing HCC metastasis.

## Materials and methods

### Clinical samples

We obtained 25 cases of the carcinoma tissues and tumor-adjacent tissues from HCC patients who experienced curative liver resection and relative serum samples from HCC patients, liver cirrhosis patients and healthy people at the affiliated cancer hospital of Tianjin Medical University. HCC tissues with distinctive tumor features were extracted from tumor nodules and stained with hematoxylin and eosin (H&E) to aid in diagnosis. Paracancerous tissues without obvious tumor features were collected at least 5 cm away from the tumor border. The above samples had the approval of the Ethics Committee of Tianjin Medical University and informed consent was obtained from HCC patients.

### Isolation and Culture of CAFs and PTFs

HCC tissues and tumor-adjacent tissues were finely cut into tiny pieces after three rinses of saline and were digested with 0.15% IV type collagenase for 30 min at 37 °C. The digested tissue fluid was centrifuged with the speed of 100 g for 5 min and the supernatant was obtained, followed by centrifuging at 300 g for 10 min. The CAFs and PTFs were washed with saline and incubated in DMEM/F-12 medium (DF-12, Gibco).

### Cell culture and cell stimulation

Huh7 and Hep3B cells were purchased from the American Type Culture Collection (ATCC, Manassas, VA, USA). According to the manufacturer’s instructions, Huh7 and Hep3B cells were cultured in Dulbecco’s modified Eagle medium (DMEM, Gibco, Gaithersburg, MD, USA) containing 10% fetal bovine serum (FBS, Gibco). All cells were tested negative for mycoplasma and cultured at 37 °C with 5% CO_2_ in a humidified incubator.

Planting the cells onto 6-well plates at the density of 2 × 10^5^ cells/well. For CCL5 stimulation in vitro, hCCL5 (HY-P70450, MedChemExpress, New Jersey, USA) in medium at the concentration of 20 ng/ml and 100 ng/ml for incubating 48 hours. For inhibitor studies, 100 nM CCR3 antagonist SB-297006 (S0129, Selleck, Houston, Texas, USA) and 100 nM CCR5 antagonist Adaptavir (S8501, Selleck) were used according to the specifications. For neutralization experiments, CM was added with 1 μg/ml hCCL5 antibody (ab9679, Abcam, Cambridge, UK) for 4 hours in advance.

### Cytokine antibody array and collection of conditioned medium

To collect the conditioned medium (CM), CAFs and PTFs were planted on 6-well plates at 2 × 10^5^ cells/well and incubated with serum-free medium for 48 h. To analyze protein expression, Human Cytokine Antibody Array C5 (AAH-CYT-G1000, RayBiotech, Atlanta, USA) was used according to the operating manual. We provided source data in supplementary information.

### Cell Migration and Invasion Assay

To assess the capacity for migration, 5 × 10^4^ cells were obtained in 100 μL DMEM without serum and plated on uncoated transwell filter inserts (8 μm pore size; Corning, USA) in 24-well plates. Adding 600 μL of medium containing 10% FBS as a chemoattractant in chamber, and then cells were incubated at 37 °C in a 5% CO_2_ incubator for 24 hours. To assess the capacity for invasion, HCC cells were seeded at density of 5 × 10^4^ cells/well on Matrigel-coated transwell filter inserts in 24-well plates (Matrigel: serum-free medium = 1:5, 50 μL/well). After incubating 48 h, the migrated or invaded cells were fixed with 4% paraformaldehyde and stained with crystal violet. The cells were counted in five random field. The experiments were repeated three times independently.

### Cell viability and wound healing assay

CAFs and PTFs were planted on 96-well plates (5 × 10^4^ cells/well) respectively. Cell proliferation was measured at 24–96 h. CCK-8 kit (HY-K0301, MedChemExpress) was applied for cell proliferation assay. Cells were grown at a high density. A linear wound was drawn across the confluent cell layer and washed twice with PBS. After incubation for period of time accompanying the treatment, size of wounds was observed and measured at 24 h.

### ELISA and flow cytometry

Human CCL5 concentration in the serum of the HCC patients, liver cirrhosis patients and healthy people as well as in the CM of CAFs and PTFs were determined with CCL5 ELISA kit (P13501, RayBiotech) under the guidance of instruction. The following antibodies were used for cytometry: anti-CD105-FITC (800505, Biolegend, San Diego, CA, USA), anti-CD73-APC (344005, Biolegend), anti-CD90-FITC (328170, Biolegend), anti-CD44-FITC (338803, Biolegend), anti-CD34-FITC (343503, Biolegend), anti-CD45-FITC (982316, Biolegend), anti-HLA-DR-FITC (980402, Biolegend) and anti-CD31-APC (303115, Biolegend). The cells staining was performed under manufacturer’s protocol and identified by a flow cytometer (FACSCanto II, BD, USA). FlowJo software (Version X; TreeStar, Ashland, OR, USA) was used to data analysis.

### RT-PCR and western blotting

Trizol reagent (Ambion, Austin, TX, USA) and the First Strand cDNA Synthesis Kit (Thermo Fisher Scientific) were used to extracted total RNA and reversed to cDNA. Quantitative real-time PCR using the SYBR Green mix (Applied Biosystem, USA) was then performed with Light Cycler® 96 Real-time PCR System. The data were calculated and analyzed by the 2^–ΔΔCt^ method. The relevant primers were listed in Table S1.

The BCA protein assay Kit (Pierce, USA) was used to determine the concentration of protein. Primary antibodies associated in this study were described as follows: anti-E-cadherin (1:1000, 20874-1-AP, Proteintech), Vimentin (1:1000, 10366-1-AP, Proteintech), anti-ZEB1 (1:1000, #70512, CST), anti-MMP2 (1:1000, #40994, CST), anti-MMP9 (1:1000, #13667, CST), anti-CCR1(1:500, ab233832, Abcam), anti-CCR3 (1:500, ab32512, Abcam), anti-CCR5 (1:500, 17476-1AP, Proteintech), anti-HIF1α (1:1000, #36169, CST), anti-HIF1α-OH402 (1:1000, ab72775, Abcam), anti-HIF1α-OH564 (1:1000, #3434, CST), anti-Ubiquitin (1:1000, #3936, CST), anti-pVHL (1:1000, #68547, CST), anti-PHD1 (1:1000, 12984-1-AP, Proteintech), anti-PHD2 (1:1000, 19886-1AP, Proteintech), anti-PHD3 (1:1000, 18325-1-AP, Proteintech). Secondary antibodies were as follows: HRP-conjugated anti-rabbit IgG (#93702, CST) or anti-mouse IgG (#58802, CST). Immobilon Western Chemiluminescent HRP Substrate (Merck Millipore, Billerica, MA, USA) was applied to visualize antibody complexes.

### Immunohistochemistry and Immunofluorescence staining

Tissue microarray slides including 108 HCC specimens were obtained from Alenabio (Xian, China). Immunostaining of tissue microarray slides, liver carcinoma, and tumor-adjacent tissues were performed with anti-ZEB1 antibody (#70512, CST, Danvers, MA, USA) and anti-α-SMA (14395-1-AP, Proteintech, Chicago, USA) respectively as described previously [10]. The staining scores were determined in terms of the proportion of positive cells and intensity.

Immunofluorescence were performed with primary antibodies against α-SMA (1:100, 14395-1-AP, Proteintech), E-cadherin (1:100, 20874-1-AP, Proteintech), Vimentin (1:100, 10366-1-AP, Proteintech), CD31 (1:3200, #3528, CST), HIF1α (1:3200, D43B5, CST), ZEB1 (1:1600, #70512, CST) followed by incubation with Alexa Fluor 555 goat antirabbit IgG (H + L) and Alexa Fluor 488 goat anti-Mouse IgG (H + L) (1:200, A21428; 1:200, A11029, Invitrogen, California, USA) as secondary antibodies.

### Chromatin immunoprecipitation (ChIP) Assay

SimpleChIP^®^ Plus Sonication Chromatin IP Kit (#56383, CST) was used for Chromatin immunoprecipitation (ChIP) assays. In short, the HCC cells were pretreated by 1% formaldehyde and incubated at 37 °C for 20 min. To obtain 200–300 bp chromatin fragments, cell lysates were sonicated and then immunoprecipitated with anti-HIF1α (#36169, CST) or normal IgG as control. The purified target genes were analyzed by quantitative real-time PCR and the data were processed by the 2^–ΔΔCt^ method. Relative amplification primers were listed in Table S1.

### Co-Immunoprecipitation (Co-IP) Assay

The HCC cells were treated by lysis buffer with complete protease inhibitors and incubated with 50 μl Protein G Magnetic Beads (#70024, CST) that were binded with the corresponding antibodies at 4 °C overnight. The beads were washed five times and centrifugated at 13,000 rpm, and the immunoprecipitates were conducted by Western blot analysis. The relevant primary antibodies were listed in Western Blotting.

### Plasmids, virus infection, generation of stable cell lines

The siRNA plasmids targeting human CCL5, CCR3, CCR5, HIF1α, and ZEB1 and overexpression plasmids targeting human HIF1α and ZEB1 were obtained from Genechem Corporation (Shanghai, China). Using the Lipofectamine 3000 reagent (Invitrogen) to perform the transfection of overexpression assay. For virus infection, these siRNA plasmids were cloned into lenti-CRISPR V2 vectors (Addgene, Watertown, MA, USA). HCC cells and CAFs were infected with lentivirus mixed with 10 μg/ml polybrene (Sigma-Aldrich, St.Louis, MO, USA). Puromycin (Sigma-Aldrich) at the concentration of 2 mg/ml was added to screen the transfected cells for generating stable cell lines.

### Animal experiment

All animal studies got approval by the Institutional Animal Care and Use Committee at Tianjin Medical University. Five-week-old female non-obese diabetic/severe combined immunodeficiency (NOD/SCID) mice were purchased from the Animal Institute of Peking Union Medical College. Five animals were randomly assigned to each group for the experiments and the sample size was estimated based on the xenograft assays in literature. For subcutaneous xenograft experiments, different cells were subcutaneously injected into the flanks of NOD/SCID mice (Huh7: CAFs or PTFs = 2 × 10^6^: 4 × 10^5^ cells/mouse). The animals were sacrificed after 40 days and tumor weight was evaluated. Measurement and data processing were carried out in confidential conditions. The lungs of mice were isolated and fixed with Bouin’s liquid. The number of lung metastatic nodules was counted. H&E staining was performed to mice lungs for histological assessment. For the survival assay, we subcutaneously injected Huh7 cells alone or accompanied with CAFs or PTFs into the flanks of NOD/SCID mice. (Huh7: CAFs or PTFs = 2 × 10^6^: 4 × 10^5^ cells/mouse). The subcutaneously injected mice were fed conventionally until their death, recording the date.

### Statistical analysis

Each experiment was strictly designed and carefully performed in triplicate, and the data were showed as the mean and standard deviation (mean ± SD). Statistical analysis was processed by using IBM SPSS Statistics 19.0 software and GraphPad Prism 9.0 software. All tests of significance were two-sided. The appropriate statistical tests were chosen for different data types. The Kolmogorov–Smirnov test was used to evaluate the normal distribution. Student *t-*test and one-way ANOVA were applied to analyze statistical significance when the data met a normal distribution. For non-normal distribution data, significant differences between the two groups were analyzed by Wilcoxon signed-rank test. The F-test verified that the variance between experimental groups was similar. χ2 test was performed to elucidate the relationship between ZEB1 expression and clinicopathologic status. The survival curve was evaluated using a log-rank test. *P* < 0.05 was considered statistically significance.

## Results

### CAFs are isolated and identified from HCC tissues

**Fig. 1:**
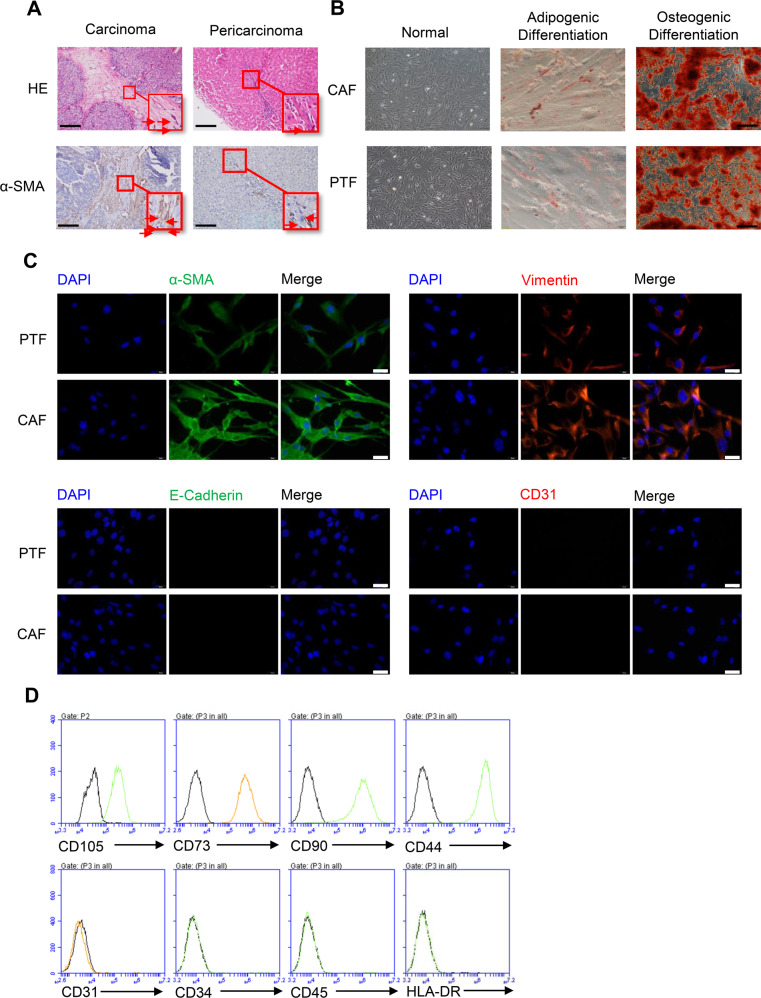
Identification and differentiation potential of CAFs isolated from HCC tissues. **A** HCC tissues and matching tumor-adjacent tissues were subjected to H&E staining respectively (upper); IHC images showed α-SMA expression respectively (lower). The red arrows indicated α-SMA positive cells. Scale bar: 100 μm. **B** The images showed representative morphology of CAFs and PTFs in vitro. The isolated CAFs and PTFs were treated with adipogenic induction and stained by Oil Red O. And CAFs and PTFs were treated with osteogenic induction and stained by Alizarin Red S. Scale bar: 100 μm. **C** Immunofluorescence staining of CAFs and PTFs showed α-SMA, Vimentin positive expression and E-cadherin, CD31 negative expression. Scale bar: 100 μm. **D** Flow cytometric analysis showed CAFs were positive for CD105, CD73, CD90, and CD44, negative for CD31, CD34, CD45, and HLA-DR.

As the important cellular components in the tumor microenvironment, CAFs overexpressed α-smooth muscle actin (α-SMA). To illustrate the role of CAFs in HCC progression, we detected the α-SMA expression in HCC tissues and matching paracancerous tissues using immunohistochemical staining. The result showed more activated fibroblasts accumulated in HCC tissues than in corresponding paracancerous tissues (Fig. 1A). Therewith, CAFs and PTFs were isolated and cultured from liver cancer tissues and paracancerous tissues and had the potential to differentiate into adipocytes and osteoblasts (Fig. 1B). We analyzed the fibroblasts marker α-SMA and the mesenchymal cells marker Vimentin to characterize the isolated CAFs and PTFs. We observed positive expression of α-SMA and Vimentin as well as negative expression of E-cadherin and CD31, which indicated that the isolated cells have fibroblast characteristics, with no epithelial cells or endothelial cells contamination (Fig. 1C). We also found that CD105, CD73, CD90, CD44 were positive, and CD31, CD45, CD34, and HLA-DR were negative, demonstrating that the CAFs have the characteristic of mesenchymal cells (Fig. 1D). In addition, CAFs had stronger proliferate and migrate capabilities than PTFs (Fig. S1A and S1B).

### CAFs boost the metastasis of HCC cells

**Fig. 2:**
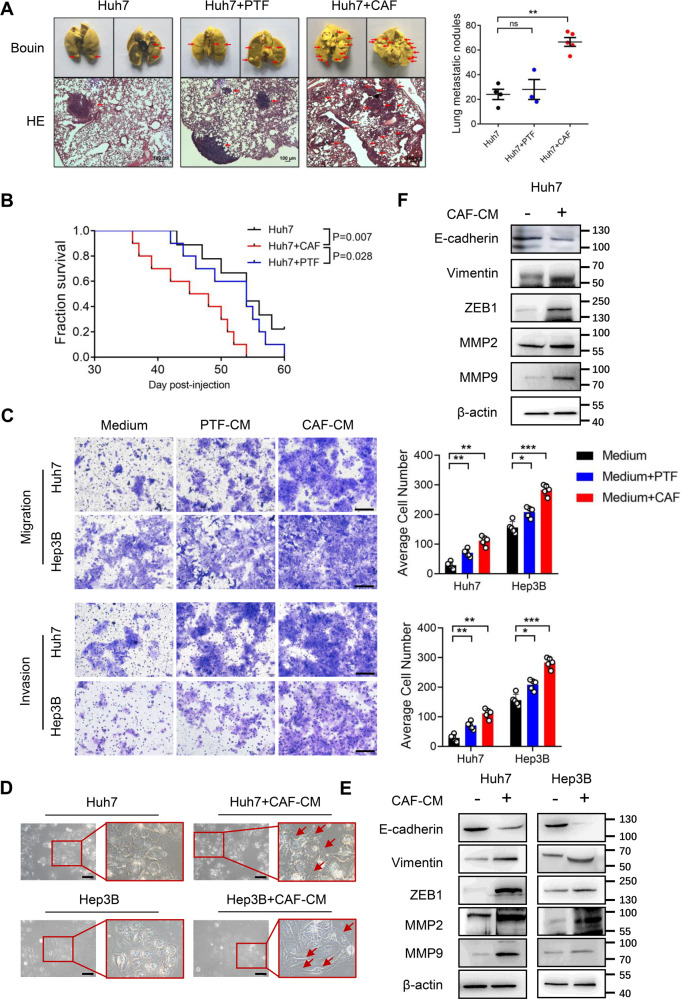
The effect of CAFs on HCC metastasis. **A** CAFs facilitated lung metastasis of liver cancer in vivo. We subcutaneously injected Huh7 cells treated with CAFs or PTFs into the flanks of NOD/SCID mice (*n* = 5). Bouin’s liquid staining showed the pulmonary metastatic nodules (red arrows) from the subcutaneous xenograft tumors of mice (upper); Representative images of H&E staining showed tumor distribution (red arrows) in both lungs (lower). Scale bar: 100 μm. **B** Kaplan-Meier plot showed overall survival of subcutaneously xenograft mice in Huh7 group or Huh7 cells treated with CAFs or PTFs group (*n* = 10). *p* = 0.007, compared with Huh7 group; *p* = 0.028, compared with Huh7+PTF group. **C** The metastasis ability of Huh7 and Hep3B cells treated with the CAF-CM or PTF-CM were detected by transwell assay. Scale bar: 50 μm. **D** Morphological changes of Huh7 and Hep3B cells treated with the CAF-CM were observed using the microscope. Scale bar: 50 μm. **E** and **F** The expression of EMT markers in Huh7 and Hep3B cells pretreated with the CAF-CM and subcutaneous tumor tissues was detected by western blotting. *N* = 5 per group. Mean ± SD, *p < 0.05, **p < 0.01, ***p < 0.001, ns: no significance, compared with the tumor control group.

To clarify the effect of CAFs on HCC progression, experimental mice were injected subcutaneously on the flank with Huh7 cells alone or co-cultured with CAFs or PTFs to establish the subcutaneous xenografts. The visible tumors were approximately formed 14 days after injection. Compared with Huh7 cells injection group, both groups that were injected with Huh7 cells containing CAFs or PTFs showed stronger tumorigenic potential, but there was no significant difference in tumor weight of the three groups at the end of the experiment (Fig. S2A). However, we monitored that all mice that formed subcutaneous tumors developed lung metastases. Moreover, the size and number of lung metastatic nodules in the Huh7 and CAFs injection group were significantly increased compared to the Huh7 cells injected alone, whereas PTFs displayed no prominent influence on the lung metastasis of Huh7 cells (Fig. 2A). Consistent with the above results, H&E staining also showed more diffuse distribution of lung metastatic nodules in the group injected with Huh7 and CAFs (Fig. 2A). Besides that, we also investigated whether survival time of subcutaneous transplanted mice was affected by CAFs. Mice in the Huh7 and CAFs group began to die 37 days after tumor formation. The mice injected with Huh7 and CAFs survived shorter than the other two groups (Fig. 2B). Our results suggested that CAFs facilitated the spread of hepatocarcinoma to both lungs, thereby affecting the tumor progression and prognosis.

Next, we evaluated the metastasis capacity of Huh7 and Hep3B cells underwent treatment of the CAF-CM or PTF-CM in vitro using transwell assay. Our studies showed that both Huh7 and Hep3B cells indicated stronger metastasis ability when treated with CAF-CM or PTF-CM, and CAFs had more pronounced influence on the metastasis capacity of HCC cells than PTFs (Fig. 2C). Meanwhile, the results of migration were verified by wound healing assay (Fig. S2B). Interestingly, the morphology of Huh7 and Hep3B cells changed from flat epithelial cells to spindle-like stromal cells, on which many pseudo-footed protrusions were formed when incubated with the CM of CAFs (Fig. 2D). Therefore, we further explored the induction of EMT by CAFs in Huh7 and Hep3B cells. Western blotting results showed that the expression of Vimentin and ZEB1 was markedly increased when Huh7 or Hep3B cells were treated with the CAF-CM, meanwhile, MMP2 and MMP9 were also significantly increased. In contrast, the expression of epithelial cell marker E-cadherin was down-regulated by CAF-CM (Fig. 2E). An analogous phenomenon was observed when the subcutaneous tumor tissues were extracted for Western blotting (Fig. 2F). The results demonstrated that CAFs induced EMT to facilitate HCC cells metastasis.

### CCL5 in the CM of CAFs regulates HCC metastasis

**Fig. 3:**
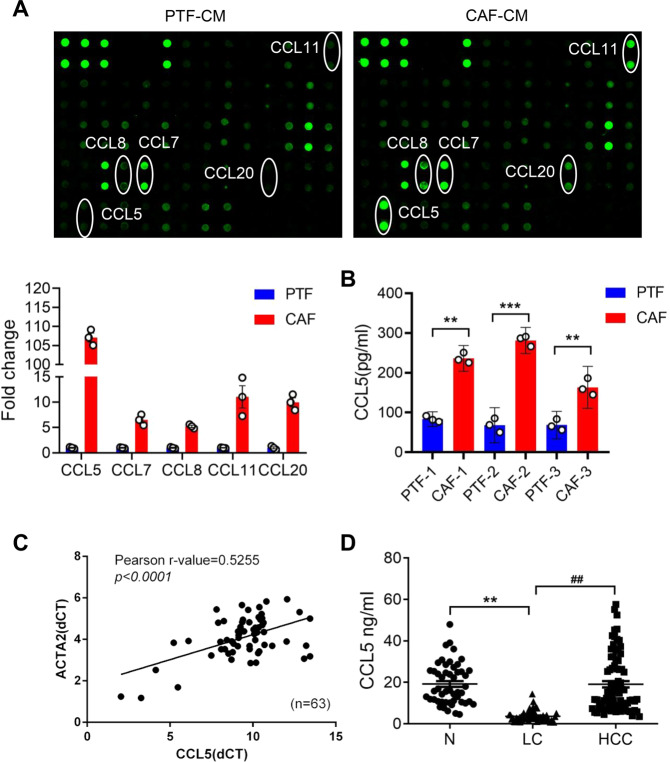
Differential analysis of secreted proteins in CAFs and PTFs. **A** The chemokine expression of the CAF-CM and PTF-CM was examined by the cytokine array. The expression of CCL5, CCL7, CCL8, CCL11 and CCL20 was significantly increased (highlighted with a circle). **B** The expression of CCL5 was measured in the CM of CAFs and PTFs from three HCC patients by ELISA. Mean ± SD, **p < 0.01, ***p < 0.001, compared with the PTF group. **C** Pearson correlation analysis showed that CCL5 was positively correlated with the expression of ACTA2 in 63 liver cancer samples (*r* = 0.5255, *p* < 0.0001). **D** The CCL5 serum expression level of HCC patients, liver cirrhosis patients and healthy person was detected by ELISA. *N* = 3 per group. Mean ± SD, **p < 0.01, compared with the healthy person group; ##*p* < 0.01, compared with the LC group.

As an important component of tumor microenvironment, CAFs induce EMT to enhance the abilities of tumor cell invasion and metastasis by secreting a variety of growth factors or chemokines [11]. To discover the important excreted factor in the CM of CAFs that promoted metastasis, we examined cytokine level in the CM of CAFs and PTFs. The expression of CCL5, CCL7, CCL8, CCL11, and CCL20 in CAF-CM was significantly higher than PTF-CM, especially CCL5 expression was the most significant (Fig. 3A). At the same time, the increased protein level of CCL5 in CAFs was examined by ELISA (Fig. 3B). In addition, we observed that CCL5 was positively correlated with ACTA2 in HCC clinical samples (*n* = 63) (Fig. 3C). In order to investigate the role of CCL5 during HCC progression, we collected three groups of clinical serum from liver cancer patients, liver cirrhosis patients and healthy person to detect CCL5 expression. As we expected, the CCL5 expression in serum of HCC patients was more obviously increased than that of liver cirrhosis patients, while it was similar to that of healthy person (Fig. 3D). Therefore, we speculated that CCL5 might be one of the important regulatory factors for CAFs to promote HCC metastasis.

**Fig. 4:**
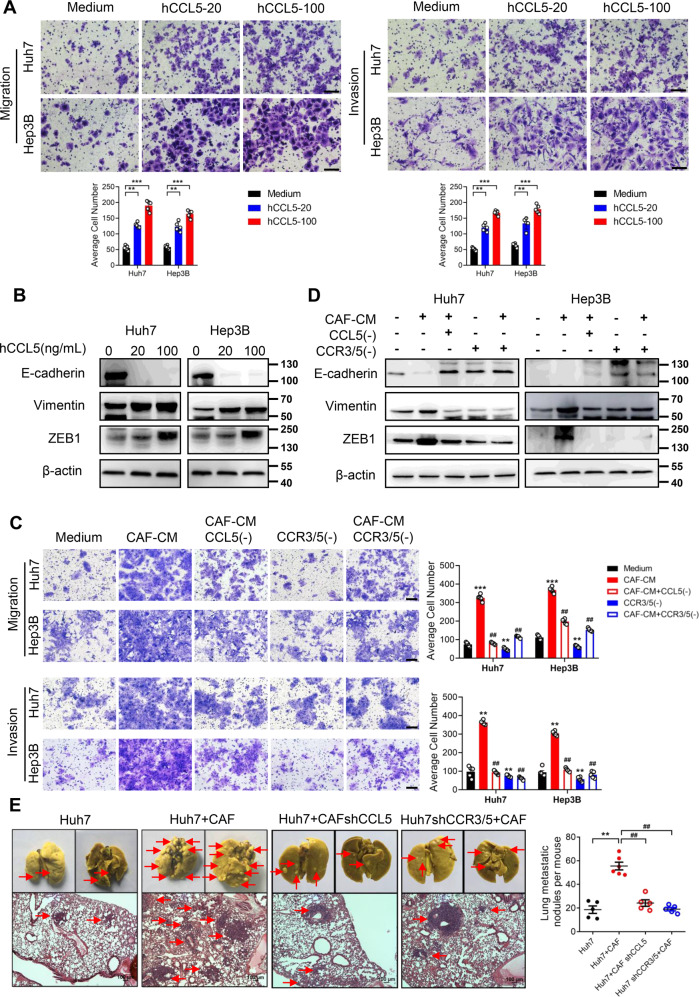
The effect of CCL5 derived from CAFs on HCC metastasis. **A** The migration and invasion abilities of Huh7 and Hep3B cells treated with hCCL5 (20 ng/ml and 100 ng/ml) were assessed by transwell assay. Scale bar: 50 μm. **B** The expression of EMT markers in Huh7 and Hep3B cells treated with hCCL5 (20 ng/ml and 100 ng/ml) was examined by western blotting. **C** Transwell assay was used to access the metastasis ability of Huh7 and Hep3B cells underwent CAF-CM incubated with a CCL5 neutralizing antibody or CCR3/CCR5 antagonists. Scale bar: 50 μm. **D** The protein level of EMT markers in Huh7 and Hep3B cells pretreated by the CAF-CM, a CCL5 neutralizing antibody or CCR3/CCR5 antagonists was accessed by western blotting. **E** NOD/SCID mice were injected subcutaneously on the flanks with Huh7 cells, Huh7 cells mixed with CAFs, Huh7 cells mixed with CCL5 knockdown (shCCL5) CAFs or CCR3/5 knockdown (shCCR3/5) Huh7 cells mixed with CAFs (*n* = 5). Bouin’s liquid staining showed the pulmonary metastatic nodules (red arrows) from the subcutaneous xenograft tumors of mice (upper); H&E staining showed tumor distribution (red arrows) in the lung (lower). Scale bar: 100 μm. *N* = 5 per group. Mean ± SD, **p < 0.01, ***p < 0.001, compared with the tumor control group. ##*p* < 0.01, compared with the CAF-CM or Huh7+CAF group.

Next, we turned our attention to clarify whether CCL5 derived from CAFs was a potential promoter of metastasis in HCC cells. Different concentrations of hCCL5 increased both migration and invasion abilities of Huh7 and Hep3B cells (Fig. 4A), and induced EMT of HCC cells by promoting the expression of Vimentin and ZEB1 and repressing the expression of E-cadherin (Fig. 4B). Consistently, the CCL5 neutralizing antibody dramatically inhibited the CAFs induced migration, invasion or EMT of Huh7 and Hep3B cells (Fig. 4C, D). And we also verified the results of migration using wound healing assay (Fig. S3A and S3B).

As we all know, the binding of CCL5 to the corresponding receptors including CCR1, CCR3 and CCR5 regulates the activation of downstream signaling. Therefore, we examined specific receptors for CCL5 in Huh7 and Hep3B cells pretreated by CAF-CM. RT-PCR showed that the CAF-CM treatment markedly increased the expression of CCR3 and CCR5 in Huh7 and Hep3B cells, but no effect on the expression of CCR1 (Fig. S4A). Meanwhile, Western blotting was performed to further verify related receptors levels, and then observed consistent results (Fig. S4B). Therewith, we investigated whether inhibiting the activation of CCR3 and CCR5 affects CAFs induced HCC cells metastasis. As expected, CCR3/CCR5 antagonists inhibited CAF-CM-induced metastasis or EMT of Huh7 and Hep3B cells (Fig. 4C, D). The wound healing assay also exhibited consistent results under the same conditions (Fig. S3B).

To further verify the role of CCL5 and related receptors in CAFs inducing HCC metastasis in vivo, we successfully knocked down CCL5 in CAFs and CCR3/5 in Huh7 cells. ELISA demonstrated that the knockdown of CCL5 in CAFs was effective (Fig. S5A). Western blotting confirmed that knocking down CCR3/5 in Huh7 cells was successful (Fig. S5B). By in vivo tumorigenicity model, we found that both Huh7 cells injected with CAF-shCCL5 cells and Huh7-shCCR3/5 cells injected with CAFs groups obviously decreased lung metastasis when compared with the Huh7 cells co-injected with CAFs. Consistent results were observed in H&E staining (Fig. 4E). Subsequently, the expression of Vimentin, ZEB1, and E-cadherin in four groups of subcutaneous tumors was detected by immunofluorescence. Knocking down CCL5 in CAFs or CCR3/5 in Huh7 cells could repress the induction of EMT by CAFs in Huh7 cells (Fig. S5C). These results testified that CCL5 derived from CAFs was related to hepatocarcinoma metastasis.

### HIF1α is the downstream factor of CCL5 mediated HCC metastasis

**Fig. 5:**
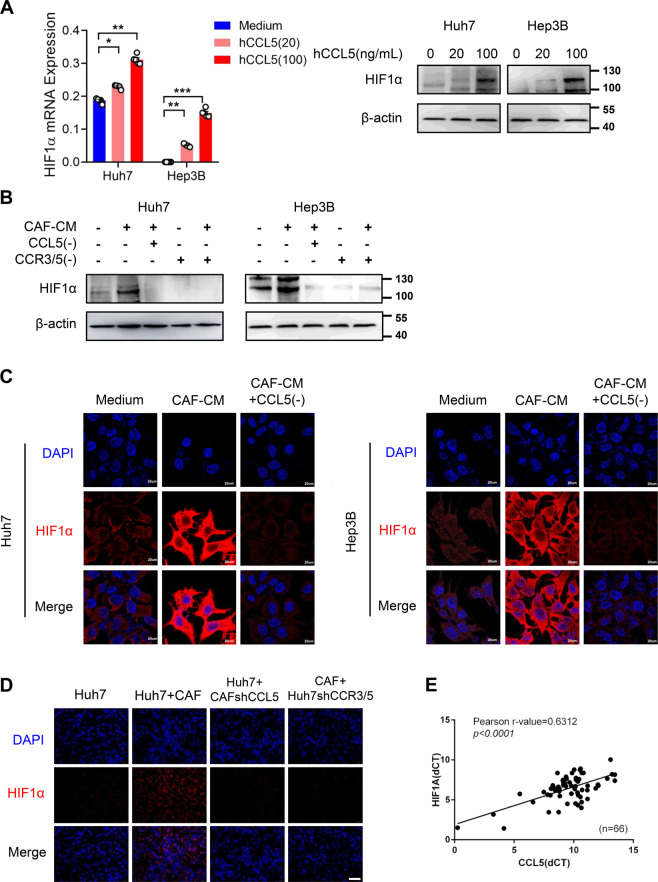
CCL5 derived from CAFs upregulates HIF1α in liver cancer cells. **A** The expression of HIF1α in Huh7 and Hep3B cells treated with hCCL5 (20 ng/ml and 100 ng/ml) was detected by RT-PCR and western blotting. **B** The protein expression of HIF1α in Huh7 and Hep3B cells treated with the CM of CAFs, a CCL5 neutralizing antibody or CCR3/CCR5 antagonists was assessed using western blotting. **C** HIF1α expression of Huh7 and Hep3B cells were tested by immunofluorescence staining and photographed under a confocal microscopy when treated with CAF-CM or CAF-CM treated with a CCL5 neutralizing antibody. Scale bar: 20 μm. **D** Immunofluorescence staining of HIF1α protein was detected in four groups of tumor samples including Huh7 cells, Huh7 cells co-injected with CAFs, Huh7 cells co-injected with shCCL5 CAFs and shCCR3/5 Huh7 cells co-injected with CAFs. Scale bar: 100 μm. **E** Pearson correlation analysis showed that HIF1A was positively correlated with the expression of CCL5 in 66 liver cancer samples (*r* = 0.6312, *p* < 0.0001). *N* = 5 per group. Mean ± SD, *p < 0.05, **p < 0.01, ***p < 0.001, compared with the tumor control group.

Hypoxic microenvironment is an important factor inducing tumor metastasis, and HIF1α plays a crucial role in the regulation of hypoxia. To further elucidate the molecular mechanism of CCL5-regulated HCC metastasis, the effect of CCL5 on HIF1α expression was investigated in Huh7 and Hep3B cells. We testified the up-regulation of HIF1α mRNA and protein expression in Huh7 and Hep3B cells when treated with hCCL5 (Fig. 5A), and we also verified the promoting effect of CAF-CM on HIF1α and the effect was abrogated when the CAF-CM was treated with CCL5 neutralizing antibody and CCR3/5 antagonists (Fig. 5B), indicating that the influence of CAFs on HIF1α was related to the presence of CCL5 in the CAF-CM. Simultaneously, immunofluorescence staining exhibited that HIF1α expression was elevated and accumulated in the nucleus in Huh7 cells or Hep3B cells treated with CAF-CM, and this phenomenon was repressed by CCL5-neutralizing antibody (Fig. 5C). And then the consistent results were confirmed in subcutaneous tumor samples (Fig. 5D). And simultaneously, we also observed that HIF1α was positively correlated with CCL5 in HCC patients (*n* = 66) (Fig. 5E). The above results suggested that CAFs up-regulated the HIF1α expression in liver cancer by secreting CCL5.

### The regulatory effect of HIF1α in HCC metastasis induced by CAFs

**Fig. 6:**
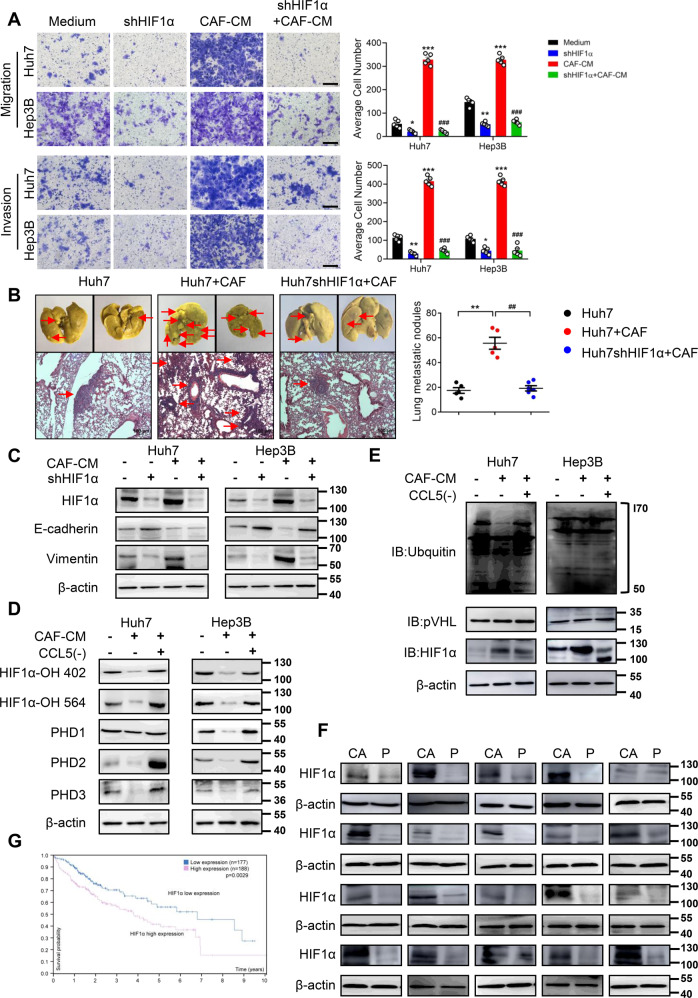
The effect of HIF1α on CCL5-induced HCC metastasis. **A** The CAF-CM induced migration and invasion of Huh7 and Hep3B cells were abrogated after knockdown of HIF1α. Scale bar: 100 μm. **B** NOD/SCID mice were injected subcutaneously on the flanks with Huh7 cells or HIF1α knockdown (shHIF1α) Huh7 cells co-injected with CAFs (*n* = 5). Bouin’s liquid staining showed the pulmonary metastatic nodules (red arrows) from the subcutaneous xenograft tumors of mice (upper); H&E staining indicated tumor distribution (red arrows) in the lung (lower). Scale bar: 100 μm. **C** The CAF-CM induced the expression of EMT markers in Huh7 and Hep3B cells was suppressed by knockdown of HIF1α. **D** The expression of hydroxylated-HIF1α (HIF1α-OH 402 and HIF1α-OH 564) and prolyl hydroxylase (PHDs) in Huh7 and Hep3B cells treated with CAF-CM or CAF-CM by pre-incubation with a CCL5 neutralizing antibody was performed by western blotting. **E** Co-immunoprecipitation of ubiquitin and pVHL with HIF1α in Huh7 and Hep3B cells treated with CAF-CM or CAF-CM by preincubation with a CCL5 neutralizing antibody. **F** HIF1α Expression in cancer tissues and matching tumor-adjacent tissues of 20 liver cancer patients was detected by western blotting. **G** Survival analysis showed overall survival of patients with high and low levels of HIF1α (*P* = 0.0029, log-rank test, https://www.proteinatlas.org/ENSG00000100644-HIF1A/). *N* = 5 per group. Mean ± SD, *p < 0.05, **p < 0.01, ***p < 0.001, compared with the tumor control group. ##*p* < 0.01, compared with the Huh7+CAF group, ###*p* < 0.001, compared with the CAF-CM group.

To elucidate whether HIF1α was the downstream factor of CCL5 in promoting HCC metastasis, we knocked down HIF1α in Huh7 and Hep3B cells (Fig. 6C). Knocking down HIF1α suppressed the migration and invasion abilities of HCC cells alone. We also observed the effect of the CAFs-mediated metastasis was prominently restrained through HIF1α knockdown (Fig. 6A). Similarly, after HIF1α knockdown, the wound healing ability of Huh7 and Hep3B cells was weakened regardless of whether treated with the CAF-CM (Fig. S6). In accordance with the previous studies in vitro, we verified that HIF1α knockdown suppressed CAF-induced lung metastasis in NOD/SCID subcutaneous tumor model, which was also observed in H&E staining (Fig. 6B). By western blotting, we also demonstrated that CAF-induced EMT was profoundly suppressed upon HIF1α knockdown (Fig. 6C).

HIF1α showed low expression due to ubiquitination and degradation under normoxic conditions, so we speculated that CCL5 might promote the stability of HIF1α in HCC cells and then entry into the nucleus by inhibiting this pathway. Western blotting results showed that hydroxylated-HIF1α (HIF1α-OH 402 and HIF1α-OH 564) and prolyl hydroxylase (PHDs) were highly expressed in Huh7 and Hep3B cells under normoxic conditions. After the induction of CAF-CM, the expression of HIF1α-OH 402, HIF1α-OH 564 and PHDs was inhibited, and this effect disappeared when added with the CCL5 neutralizing antibody (Fig. 6D). Subsequently, Co-Immunoprecipitation (Co-IP) was used to investigate the expression of ubiquitin and pVHL. Similarly, the CAF-induced down-regulation of HIF1α ubiquitin and pVHL expression was also reversed after the addition of CCL5 neutralizing antibody (Fig. 6E), which consistently confirmed our hypothesis. Clinically, we detected HIF1α in hepatocellular carcinoma tissues and matching paracancerous tissues, and almost all samples exhibited higher HIF1α expression in tumor tissues compared with paracancerous tissues (Fig. 6F). In addition, high HIF1α expression also correlated with reduced overall survival (*p* = 0.0029) (Fig. 6G).

### CAFs promote the direct binding of HIF1α and ZEB1 to regulate HCC metastasis

**Fig. 7:**
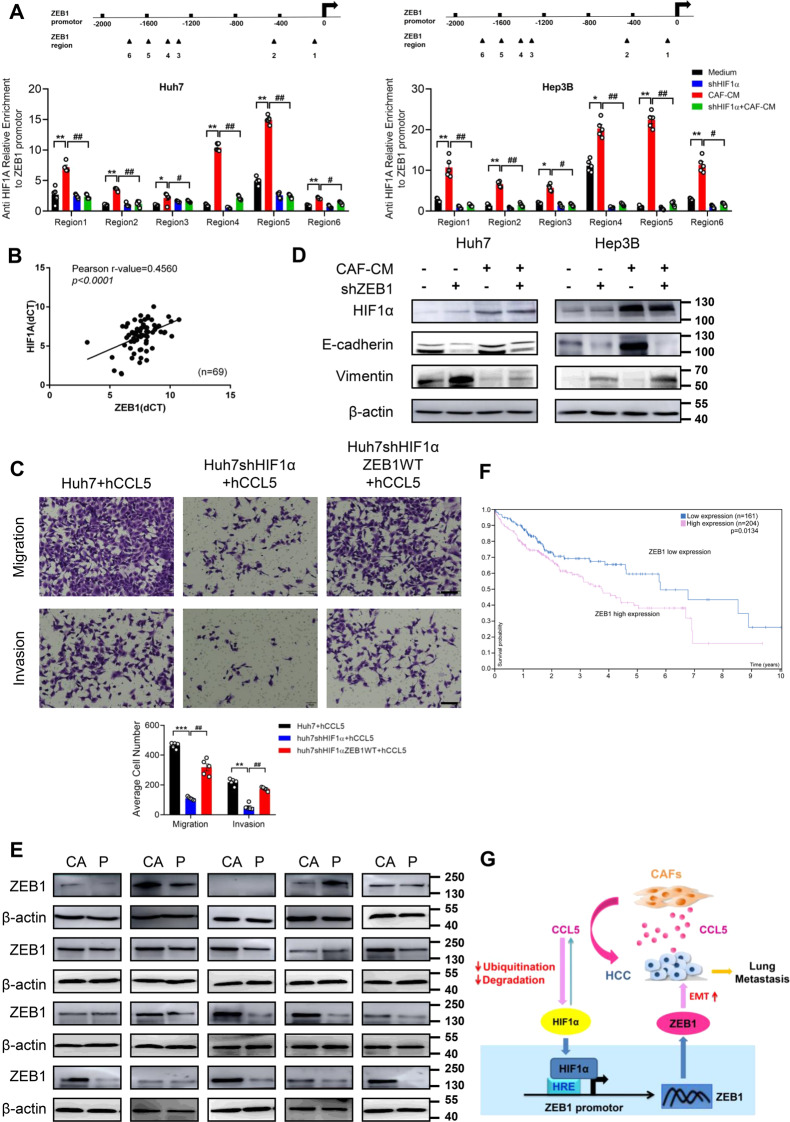
ZEB1 is a possible downstream factor of HIF1α. **A** ChIP assay detected the relative enrichments of DNA bound to ZEB1 promoter at six binding sites in Huh7 and Hep3B cells treated with CAF-CM as well as correspongding HIF1α knockdown cells. Mean ± SD, *p < 0.05, **p < 0.01, compared with the tumor control group. #*p* < 0.05, ##*p* < 0.01, compared with the CAF-CM group. **B** Pearson correlation analysis showed that HIF1A was positively correlated with the expression of ZEB1 in 69 HCC samples (*r* = 0.4560, *p* < 0.0001). **C** The migration and invasion abilities of ZEB1-overexpressing HIF1α knockdown (ZEB1-shHIF1α) Huh7 cells treated with hCCL5 (100 ng/ml) were detected by transwell assay. Scale bar: 50 μm. *N* = 5 per group. Mean ± SD, **p < 0.01, ***p < 0.001, compared with Huh7 + hCCL5 group. ##*p* < 0.01, compared with the Huh7shHIF1α + hCCL5 group. **D** ZEB1 was knocked down in Huh7 and Hep3B cells pretreated by CAF-CM or not, and the expression of HIF1α and EMT markers was detected using western blotting. **E** ZEB1 expression in cancer tissues and matched tumor-adjacent tissues of 20 liver cancer patients was detected by western blotting. **F** Survival analysis showed overall survival of patients with high and low levels of ZEB1 expression (*P* = 0.0134, log-rank test, https://www.proteinatlas.org/ENSG00000148516-ZEB1/). **G** Graphical illustration of the mechanism by which CAF-derived CCL5 inhibited the ubiquitination degradation of HIF1α and up-regulated the downstream ZEB1 to promote liver cancer metastasis.

As a significance downstream factor in the HIF1α pathway, ZEB1 plays a critical role in EMT in hepatocellular carcinoma. Knocking down HIF1α in Huh7 and Hep3B cells significantly inhibited CAF-induced ZEB1 expression (Fig. S7A). Up-regulation of ZEB1 expression in HIF1α overexpression HCC cells was also confirmed (Fig. S7B). To confirm the regulatory effect of HIF1α on ZEB1, we predicted six binding sites of Hif1α in ZEB1 promoter region by bioinformatics analysis and performed Chromatin immunoprecipitation assay. The result confirmed the relative enrichments of DNA bound to ZEB1 promoter at six binding sites were significantly increased in Huh7 and Hep3B cells pretreated by CAF-CM. On the contrary, the phenomenon was suppressed in HIF1α knockdown cells (Fig. 7A), demonstrating that ZEB1 was directly transcriptionally regulated by HIF1α. In accordance with the above research findings, we found that HIF1α expression was positively correlated with ZEB1 level in hepatocellular carcinoma specimens (Fig. 7B). This result was further confirmed by the co-localization of HIF1α and ZEB1 both in nucleus and cytoplasm of HCC tissues (Fig. S7C). To validate whether ZEB1 is a downstream regulator of HIF1α promoting liver cancer metastasis, ZEB1 was overexpressed in Huh7 cells of HIF1α knockdown. Upon HIF1α knockdown, the number of tumor cells crossing the membrane was significantly decreased, and this inhibition partially retrieved after ZEB1 overexpression (Fig. 7C). Moreover, we inhibited ZEB1 expression in Huh7 and Hep3B cells to investigate the effect on EMT. Western blotting confirmed that knocking down ZEB1 in Huh7 and Hep3B cells was effective (Fig. S7D). The results showed that ZEB1 knockdown inhibited EMT in both HCC cells and had no effect on HIF1α expression (Fig. 7D). Finally, we randomly detected the ZEB1 expression in 20 hepatocellular carcinoma patients by Western blotting, in which 15 patients had higher ZEB1 expression in HCC tissues compared with the paracancerous tissues (Fig. 7E). Clinically, HCC patients with high ZEB1 expression had obviously shorter overall survival, comparing with low ZEB1 expression (*p* = 0.0134) (Fig. 7F), and high ZEB1 expression in HCC patients was related with pathological grading (*p* = 0.0022), and TNM stage (*p* = 0.0052) (Table S2).

## Discussion

CAFs are one of the most common cell populations in the tumor stroma, which directly regulate tumor growth, progression and metastasis by affecting angiogenesis and tumor immunity [12]. A previous study had shown that CAFs were closely related to the invasion and metastasis of HCC cells [13, 14], but the mechanism of CAFs in regulating HCC metastasis was not fully clarified. In this study, we indicated that CCL5 was a crucial mediator interacting CAFs and HCC cells. CCL5 inhibited the ubiquitination degradation of HIF1α and up-regulated the downstream ZEB1 to promote liver cancer metastasis (Fig. 7G). To address this issue, we proved that CAFs enhanced the HCC metastasis by inducing EMT. Simultaneously, CAFs significantly motivated the lung metastasis of hepatocellular carcinoma cells in NOD/SCID tumor-bearing mice and shortened their survival time. These findings confirmed that CAFs had an important impact on the progression of HCC in the tumor microenvironment.

It has been reported that CAFs do not exist independently around tumors, but interact with tumor cells to promote their malignant phenotype. Tumor cells can influence the recruitment of CAFs precursors and induce normal fibroblasts into CAFs. Meanwhile, CAFs can secrete large amounts of cytokines, growth factors, and extracellular matrix proteins that create a tumor-permissive environment and motivate tumor proliferation, drug resistance, and metastasis, thus affecting tumor prognosis [15]. We found that CCL5 was the most prominent cytokine in the process of CAFs promoting liver cancer metastasis by cytokine antibody array. It has been reported that CCL5, as an inflammatory mediator, increases mainly in the acute and early stages of liver injury, and decreases as liver fibrosis gradually develops into cirrhosis in the later stages of injury [16, 17]. Our study also observed a lower level of CCL5 in the serum of patients with cirrhosis, and CCL5 expression in serum of patients increased significantly in the development from cirrhosis to hepatocellular carcinoma. While there was no statistical difference in serum CCL5 of HCC patients compared with the normal population. According to the proteinatlas HCC patient samples (ENSG00000271503CCL5), there was also no significant differences in survival probability between CCL5 high expression and CCL5 low expression patients. These results suggested that CCL5 played a crucial role in the progression of cirrhosis to liver cancer, but CCL5 might not be a specific marker of HCC. Moreover, we will further expand the clinic sample size to verify the function of CCL5 in liver cancer.

CCL5, a significant member of chemotactic cytokines, plays an important role in CAFs promoting tumor development [18, 19]. The study indicated that KLF5 promoted the invasive and metastatic abilities of tumor cells by activating the CCL5/CCR5 axis in gastric cancer-associated fibroblasts [20]. CAF-derived CCL5 was found to enhance the migratory ability of HCC cells by triggering Hedgehog pathway [5]. Similarly, we observed that CAF-derived CCL5 or exogenous hCCL5 were able to promote the metastasis ability of HCC cells. Three specific receptors of CCL5 include CCR1, CCR3, and CCR5.We also found that CAF-derived CCL5 or exogenous hCCL5 could obviously enhanced the expression of CCR3 and CCR5 in hepatocellular carcinoma cells. The use of CCL5-neutralizing antibody or CCR3/CCR5 inhibitors efficiently neutralized CAF-derived CCL5 or blocked CCR3/CCR5 activation, thereby abolishing the promotion of CCL5 on HCC metastasis. In addition, these findings in vitro were validated by in vivo results that the number of pulmonary metastatic nodules was increased by CAFs in HCC mouse model. When CCL5 or CCR3/CCR5 was inhibited, the effect of CAFs on HCC metastasis was significantly reduced. The above results theoretically confirmed the crucial role of CCL5 to regulate the relationship between CAFs and HCC metastasis.

HIF1α plays crucial roles in the regulation of energy metabolism, angiogenesis and other processes in the tumor microenvironment, enabling cancer cells to undergo EMT under metabolic stress and gaining greater proliferation and metastasis capacities. It had been reported that CAFs were involved in the regulation of tumor HIF1α signaling pathway to promote tumor progression. CAFs enhanced the EMT process by activating the MAOA/mTOR/ HIF1α signaling axis and enhanced the migration and invasion abilities of prostate cancer cells [9]. Recent research indicated that high expression of miR-224 in CAFs facilitated proliferation, EMT and metastasis of non-small cell lung cancer cells by the SIRT3/AMPK/mTOR/ HIF1α signaling pathway [21]. Here, we found that exposure to CAFs could activate HIF1α in normoxic conditions. Studies showed that exogenous hCCL5 or CAF-derived CCL5 maintained HIF1α expression and promoted it into the nucleus of HCC cells under normoxia. Knockdown of HIF1α in HCC cells significantly inhibited CAF-induced EMT and metastasis, and the number of pulmonary metastatic nodules induced by CAFs was also significantly reduced in NOD/SCID mice. In addition, CCL5 and HIF1α were positively correlated in clinical HCC samples, and high HIF1α expression was closely connection with poor prognosis in liver cancer patients. These data supported the important role of HIF1α signaling pathway in regulating CAF-derived CCL5 induced EMT and metastasis in hepatocellular carcinoma.

HIF1α is hydroxylated at p402 and p564 proline residues by prolyl hydroxylases (PHDs) because of instability under normoxic conditions, which binds to the tumor suppressor protein VHL and is rapidly degraded via the E3 ubiquitin-von Hippel-Lindau protein (pVHL) complex proteasome pathway. If HIF1α degradation pathway is inhibited, HIF1α in the cytoplasm may translocate into the nucleus and bind to downstream target genes, thereby initiating the expression of downstream genes [22]. We found that CAF-CM could significantly inhibit the expression of HIF1α-OH 402, HIF1α-OH 564 and PHDs in HCC cells, meanwhile the levels of HIF1α ubiquitin and pVHL were also suppressed. And the above inhibition could be rescinded by CCL5 neutralizing antibody. These data demonstrated that CAF-secreted CCL5 could effectively suppress the ubiquitination degradation of HIF1α in HCC cells under normoxic conditions and promote its stable expression in the nucleus as well as regulation of the target genes.

As one of the transcription factors for EMT in tumor cells, ZEB1 down-regulates the transcription of E-cadherin in the nucleus as well as up-regulates the transcription of mesenchymal marker proteins, thereby promoting tumor metastasis [23]. In addition, HIF1α had been reported to promote the expression of ZEB1 in hypoxia, and the HIF1α/ZEB1 axis enhanced cancer cells aggressiveness and distant metastasis in bladder cancer and glioblastoma [24, 25]. In our research, HIF1α regulated its transcription and expression by directly binding to ZEB1, and knockdown of ZEB1 in HCC cells effectively inhibited CAF-induced EMT and metastasis, suggesting that ZEB1 was a downstream key factor of HIF1α in the adjustment of CAF-derived CCL5 induced EMT and metastasis in HCC cells.

In conclusion, our studies reveal a novel mechanism whereby CAFs promote HCC cells invasion and metastasis in the tumor microenvironment. As a crucial regulator involving in the interaction between CAFs and liver cancer cells, CCL5 promotes HCC metastasis through activation of HIF1α/ZEB1 signaling axis. Our findings provide the cornerstones for further elucidating the mechanism of liver cancer progression and metastasis, conducing to reveal potential therapeutic targets and propose new strategies for clinical treatment of HCC.

## Supplementary information

Supplementary Information-Figure legends

Supplementary Information-Table S1

Supplementary Information-Table S2

Fig. S1

Fig. S2

Fig. S3

Fig. S4

Fig. S5

Fig. S6

Fig. S7

AJ Checklist

Data Availability Statement

The human medical ethics review sheet

## Data Availability

All data generated or analyzed during this study are included in this published article and its supplementary information files. Data sharing is not applicable to this article as no datasets were generated or analyzed during the current study. The datasets generated and analyzed during the current study are not publicly available due to unpublished but are available from the corresponding author on reasonable request.
