# Supplementary material for: Cancer associated fibroblast–derived CCL5 promotes hepatocellular carcinoma metastasis through activating HIF1α/ZEB1 axis
Source: Cell Death Dis. 2022 May 20;13(5):478. doi: 10.1038/s41419-022-04935-1 (PMC9119971; doi:10.1038/s41419-022-04935-1)
Supplement: Supplementary file 1 — Supplementary Information-Figure legends [file 41419_2022_4935_MOESM1_ESM.docx]

**Supplementary Information**

**Supplementary Legends**

Fig. S1. The difference in proliferation and migration capacity between CAFs and PTFs. (A) The proliferation of CAFs and PTFs was tested using CCK-8 assay. (B) Performing transwell assay to detect the migration of CAFs and PTFs. Scale bar: 50μm. N=5 per group. Mean ± SD, **** p < 0.0001, compared with the PTF group.

Fig. S2. CAFs facilitate the tumorigenesis and migration of liver cancer cells. (A) NOD/SCID mice were injected subcutaneously on the flanks with Huh7 cells accompanied with CAFs or PTFs and measured tumor weight after 40 days. P = 0.0808, compared with the tumor control group. (B) The migration of Huh7 and Hep3B cells treated with the CAF-CM or PTF-CM was detected using wound healing assay. Scale bar: 100μm. N=5 per group. Mean ± SD, **p < 0.01, ***p < 0.001, ns: no significance, compared with the tumor control group.

Fig. S3. CCL5 derived from CAFs promotes the migration of HCC cells. (A) The migration of Huh7 and Hep3B cells underwent hCCL5 (20ng/ml and 100ng/ml) was tested using wound healing assay. Scale bar: 100μm. (B) The migration ability of Huh7 and Hep3B cells treated with the CM of CAFs, a CCL5 neutralizing antibody or CCR3/CCR5 antagonists was detected by wound healing assay. Scale bar: 100μm. N=5 per group. Mean ± SD, *p < 0.05, **p < 0.01, ***p < 0.001, compared with the tumor control group. #p < 0.05, ##p < 0.01, compared with the CAF-CM group.

Fig. S4. The effects of hCCL5 or CAF-CM on CCR1, CCR3 and CCR5 levels in Huh7 and Hep3B cells. (A) Measuring mRNA expression of CCR1, CCR3 and CCR5 in Huh7 and Hep3B cells treated with CAF-CM by Real-time PCR. (B) Protein expression of CCR1, CCR3 and CCR5 in Huh7 and Hep3B cells treated with CAF-CM or hCCL5 (20ng/ml and 100ng/ml) was performed by western blotting. N=5 per group. Mean ± SD, *p < 0.05, **p < 0.01, compared with the tumor control group.

Fig. S5. (A) ELISA was performed to confirm the expression level of CCL5 when it was knockdown in CAFs. (B) Western blotting was performed to verify the expression level of CCR3/5 if they were knockdown in Huh7 cells. (C) Immunoﬂuorescence staining used for observing E-cadherin, Vimentin and ZEB1 protein was performed in four groups of tumor samples including Huh7 cells, Huh7 cells co-cultured with CAFs, Huh7 cells co-cultured with shCCL5 CAFs and shCCR3/5 Huh7 cells co-cultured with CAFs. Scale bar: 100μm.

Fig. S6. CAF-CM triggered migration of Huh7 and Hep3B cells was accessed by wound healing assay, which were abolished by knockdown of HIF1α. Scale bar: 100μm. N=5 per group. Mean ± SD, *p < 0.05, **p < 0.01, compared with the tumor control group. #p < 0.05, compared with the CAF-CM group.

Fig. S7. The effect of CAFs on HCC metastasis by regulating HIF1α/ZEB1. (A) Knocking down HIF1α in Huh7 and Hep3B cells, CAF-CM induced ZEB1 expression was detected using western blotting. (B) Overexpressing HIF1α in Huh7 and Hep3B cells, HIF1α and ZEB1 protein were detected by western blotting. (C) A human liver cancer specimen was detected by immunoﬂuorescence staining and photographed under a fluorescence microscopy. (D) Western blotting was performed to confirm the expression level of ZEB1 if it was knockdown in Huh7 and Hep3B cells. Scale bar: 100μm.
