# Supplementary material for: Cancer associated fibroblast–derived CCL5 promotes hepatocellular carcinoma metastasis through activating HIF1α/ZEB1 axis
Source: Cell Death Dis. 2022 May 20;13(5):478. doi: 10.1038/s41419-022-04935-1 (PMC9119971; doi:10.1038/s41419-022-04935-1)
Supplement: Supplementary file 2 — Supplementary Information-Table S1 [file 41419_2022_4935_MOESM2_ESM.docx]

**Supplementary Information**

**Supplementary Table S1**

Table S1. Primer sequences for qPCR analysis and ChIP assay

| Gene | Forward primer (5'-3') | Reverse primer (5'-3') |
| --- | --- | --- |
| CCL5 | CCAGCAGTCGTCTTTGTCAC | CTCTGGGTTGGCACACACTT |
| ACTA2 | TTCGTTACTACTGCTGAGCGTGAGA | AAGGATGGCTGGAACAGGGTC |
| CCR1 | GACTATGACACGACCACAGAGT | CCAACCAGGCCAATGACAAATA |
| CCR3 | TGGCATGTGTAAGCTCCTCTC | CCTGTCGATTGTCAGCAGGATTA |
| CCR5 | TTCTGGGCTCCCTACAACATT | TTGGTCCAACCTGTTAGAGCTA |
| HIF1A | GAACGTCGAAAAGAAAAGTCTCG | CCTTATCAAGATGCGAACTCACA |
| ZEB1 | GATGATGAATGCGAGTCAGATGC | ACAGCAGTGTCTTGTTGTTGT |
| ZEB1-32 | GGACTGGAAAGCGGAAACTT | CGAGGTGTCTGGGAGTTGG |
| ZEB1-476 | CGGGGGAGGGGGACTGGAAAGC | AGGCCTCCTGGAAACGGTGCCG |
| ZEB1-1343 | GCTGGGCCAGGCTGCTTTGCA | GGACACCTGACCTTCCGACG |
| ZEB1-1400 | CATCCCTGGAAGCTGCTCTCT | ATCCAGTGTTTACGGAGCGC |
| ZEB1-1500 | CCAGGACAATGAAACCAC | AGGAGCCCAAAGTAACAG |
| ZEB1-1731 | AACCTTGTTGCTAGGGACCG | AGTCACTTCCCATCCCGGTT |
| β-actin | CATGTACGTTGCTATCCAGGC | CTCCTTAATGTCACGCACGAT |
