# Supplementary material for: Cancer associated fibroblast–derived CCL5 promotes hepatocellular carcinoma metastasis through activating HIF1α/ZEB1 axis
Source: Cell Death Dis. 2022 May 20;13(5):478. doi: 10.1038/s41419-022-04935-1 (PMC9119971; doi:10.1038/s41419-022-04935-1)
Supplement: Supplementary file 3 — Supplementary Information-Table S2 [file 41419_2022_4935_MOESM3_ESM.docx]

**Supplementary Information**

**Supplementary Table S2**

Table S2. Correlation between ZEB1 expression level and clinic-pathological parameters of liver cancer patients

| Characteristic | Number | ZEB1 levels | | P |
| --- | --- | --- | --- | --- |
|  |  | Low | High |  |
| Age (yr) |  |  |  |  |
| ≥50  <50 | 60  48 | 22  21 | 38  27 | 0.4549 |
| Gender |  |  |  |  |
| Male  Female | 68  40 | 25  18 | 43  22 | 0.3985 |
| Pathological Grading |  |  |  |  |
| I-II  III | 81  27 | 39  4 | 42  23 | 0.0022 |
| TNM Stage |  |  |  |  |
| I-II  III-IV | 50  58 | 27  16 | 23  42 | 0.0052 |
