# Supplementary figures and images for: Cancer associated fibroblast–derived CCL5 promotes hepatocellular carcinoma metastasis through activating HIF1α/ZEB1 axis
Source: Cell Death Dis. 2022 May 20;13(5):478. doi: 10.1038/s41419-022-04935-1 (PMC9119971; doi:10.1038/s41419-022-04935-1)

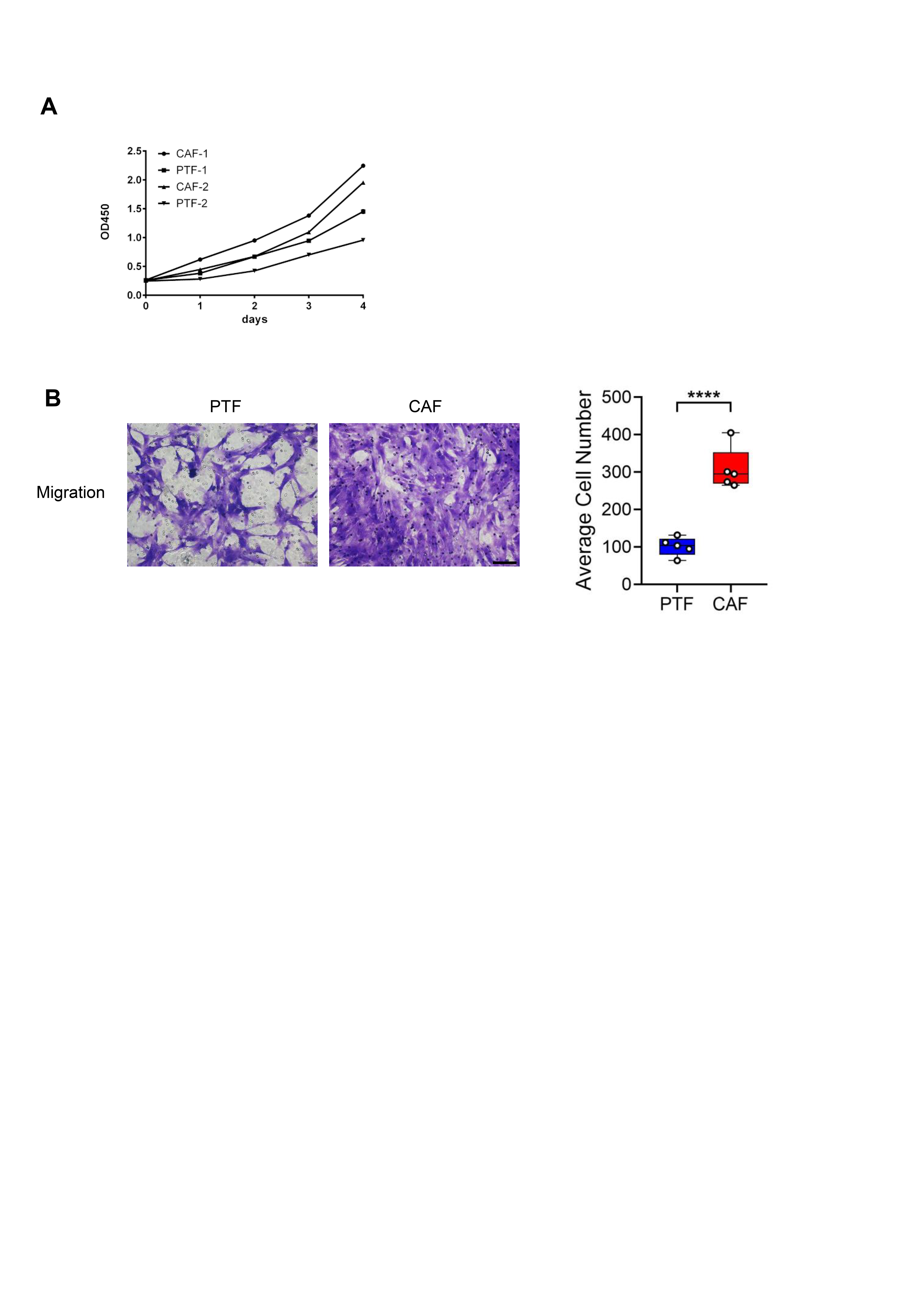

Supplement: Supplementary file 4 — Fig. S1 [file 41419_2022_4935_MOESM4_ESM.tif]

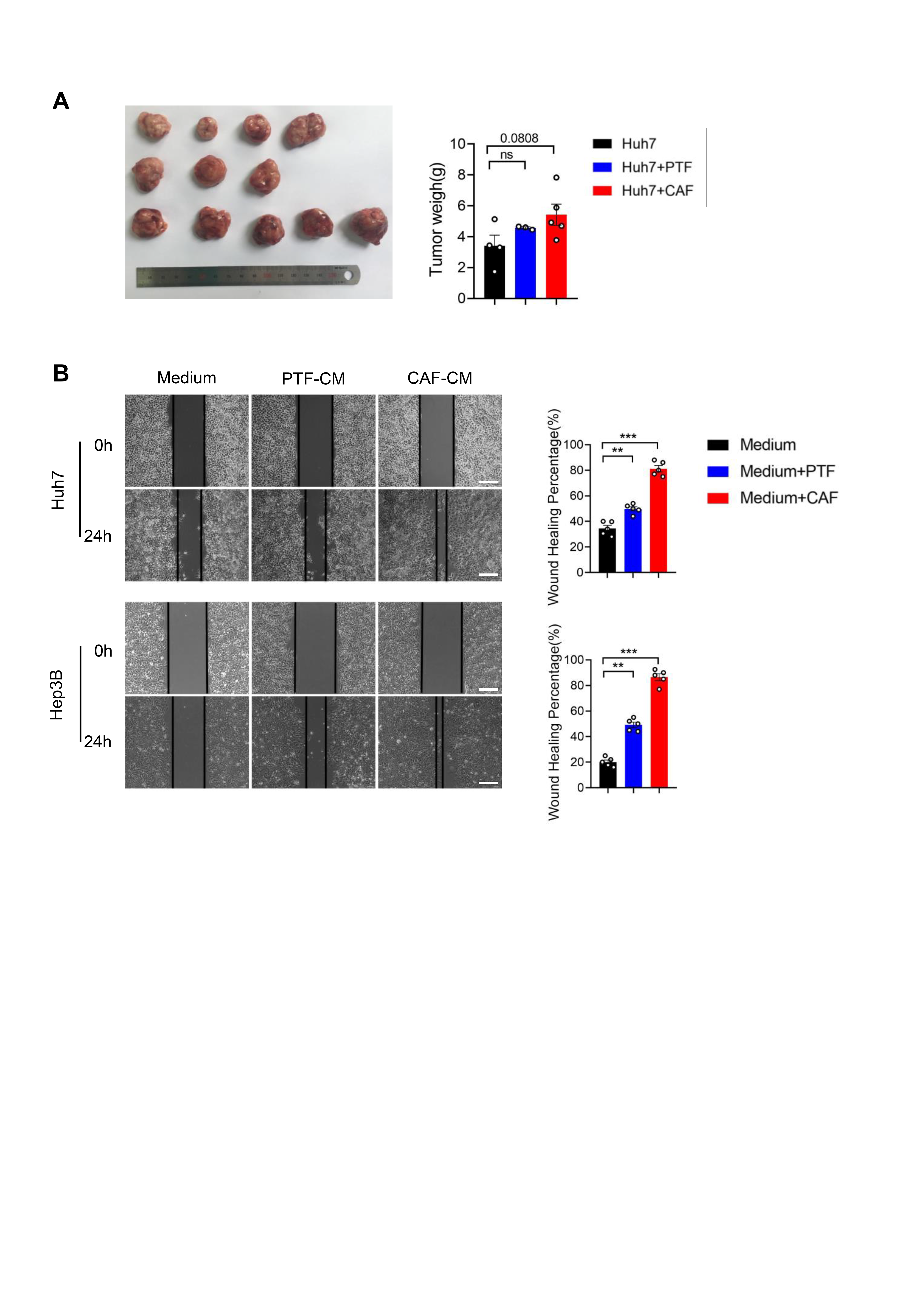

Supplement: Supplementary file 5 — Fig. S2 [file 41419_2022_4935_MOESM5_ESM.tif]

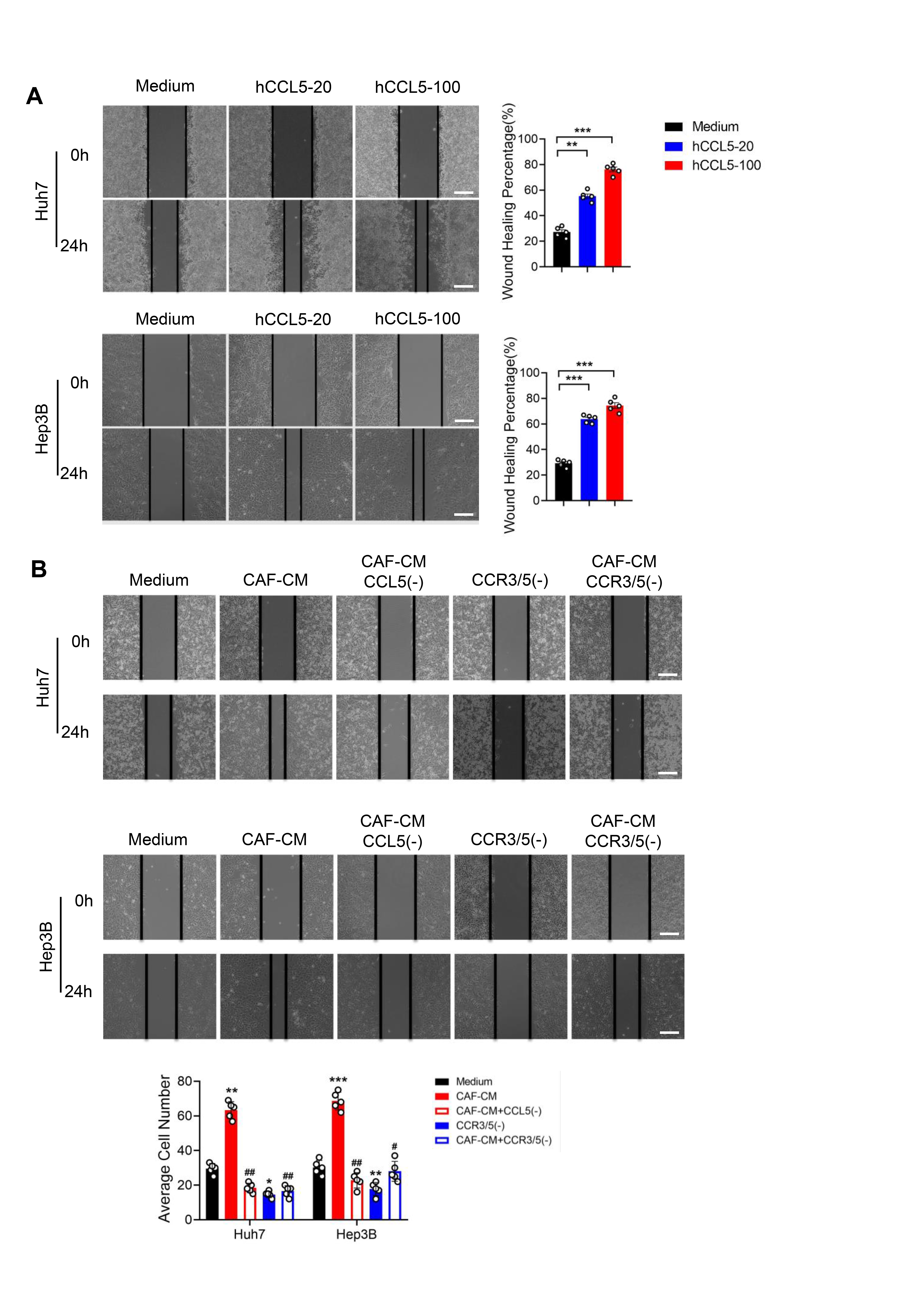

Supplement: Supplementary file 6 — Fig. S3 [file 41419_2022_4935_MOESM6_ESM.tif]

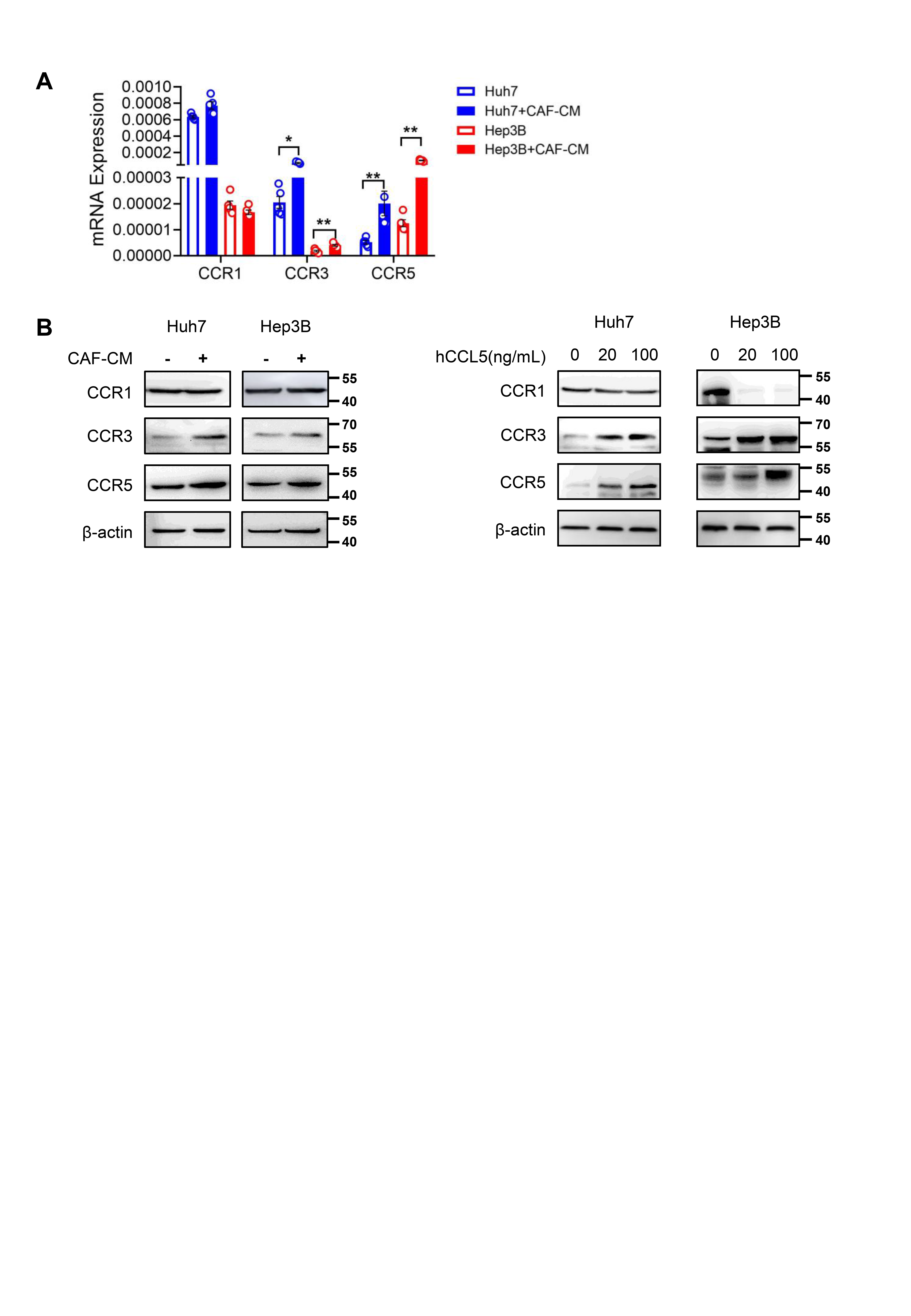

Supplement: Supplementary file 7 — Fig. S4 [file 41419_2022_4935_MOESM7_ESM.tif]

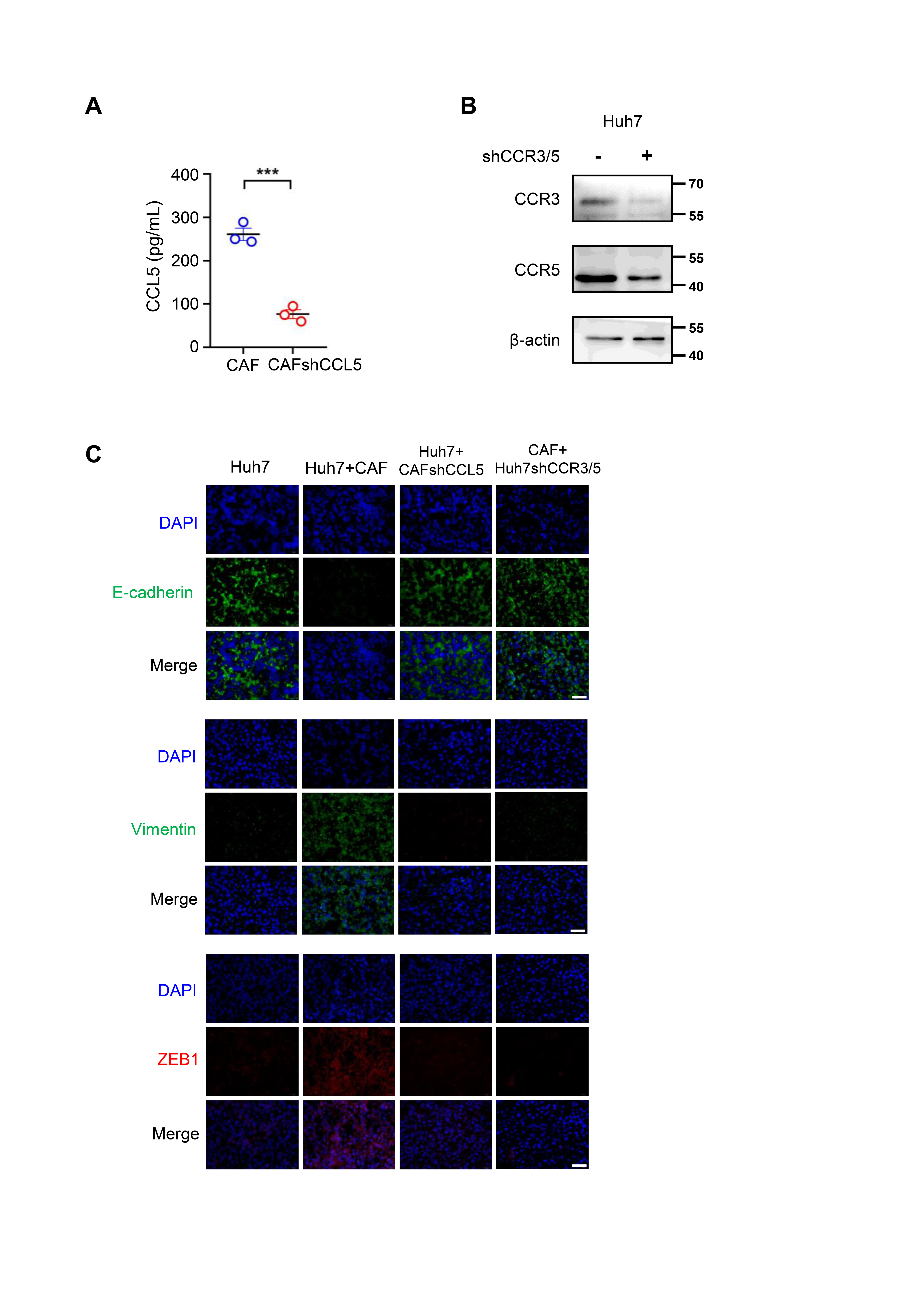

Supplement: Supplementary file 8 — Fig. S5 [file 41419_2022_4935_MOESM8_ESM.tif]

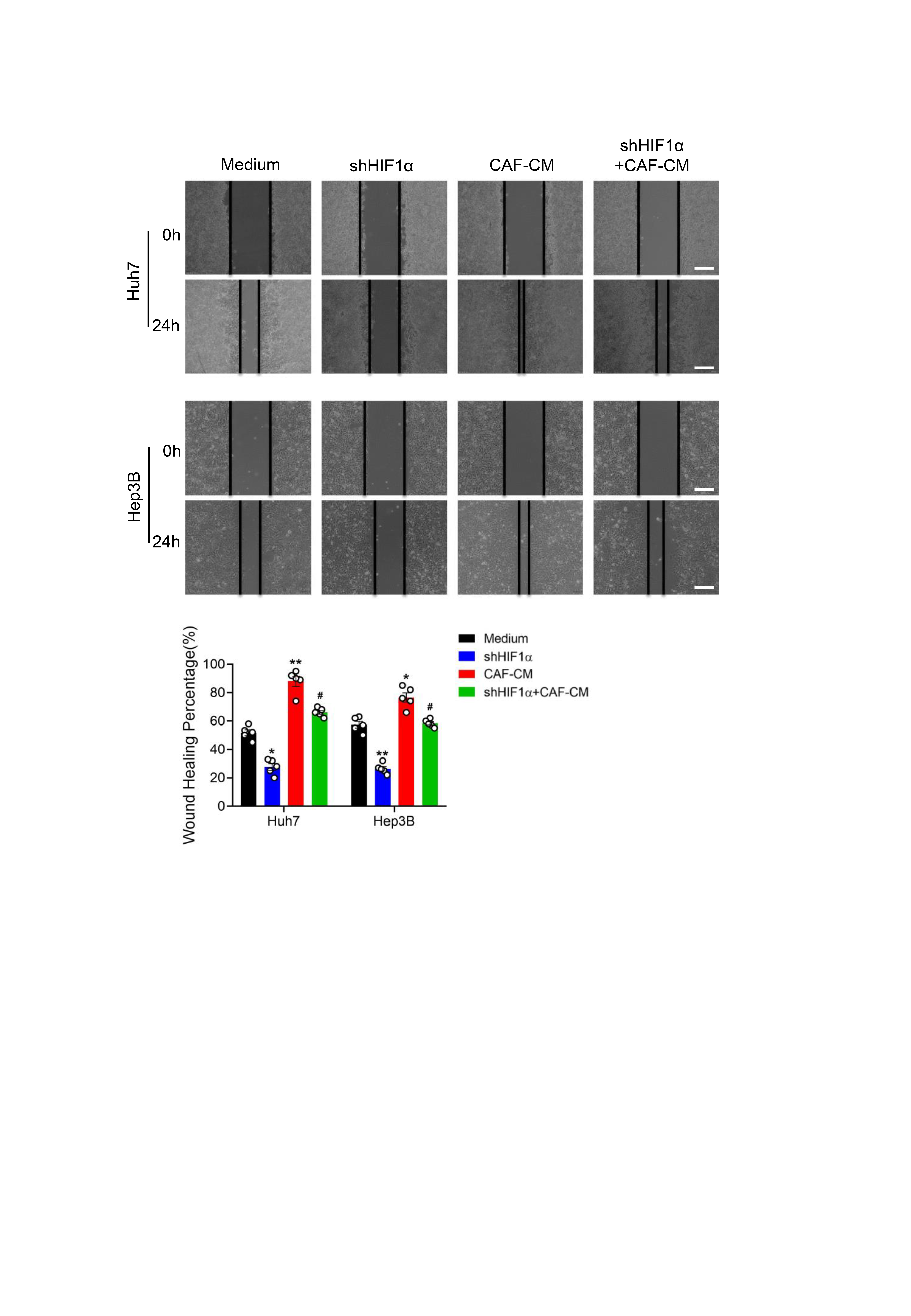

Supplement: Supplementary file 9 — Fig. S6 [file 41419_2022_4935_MOESM9_ESM.tif]

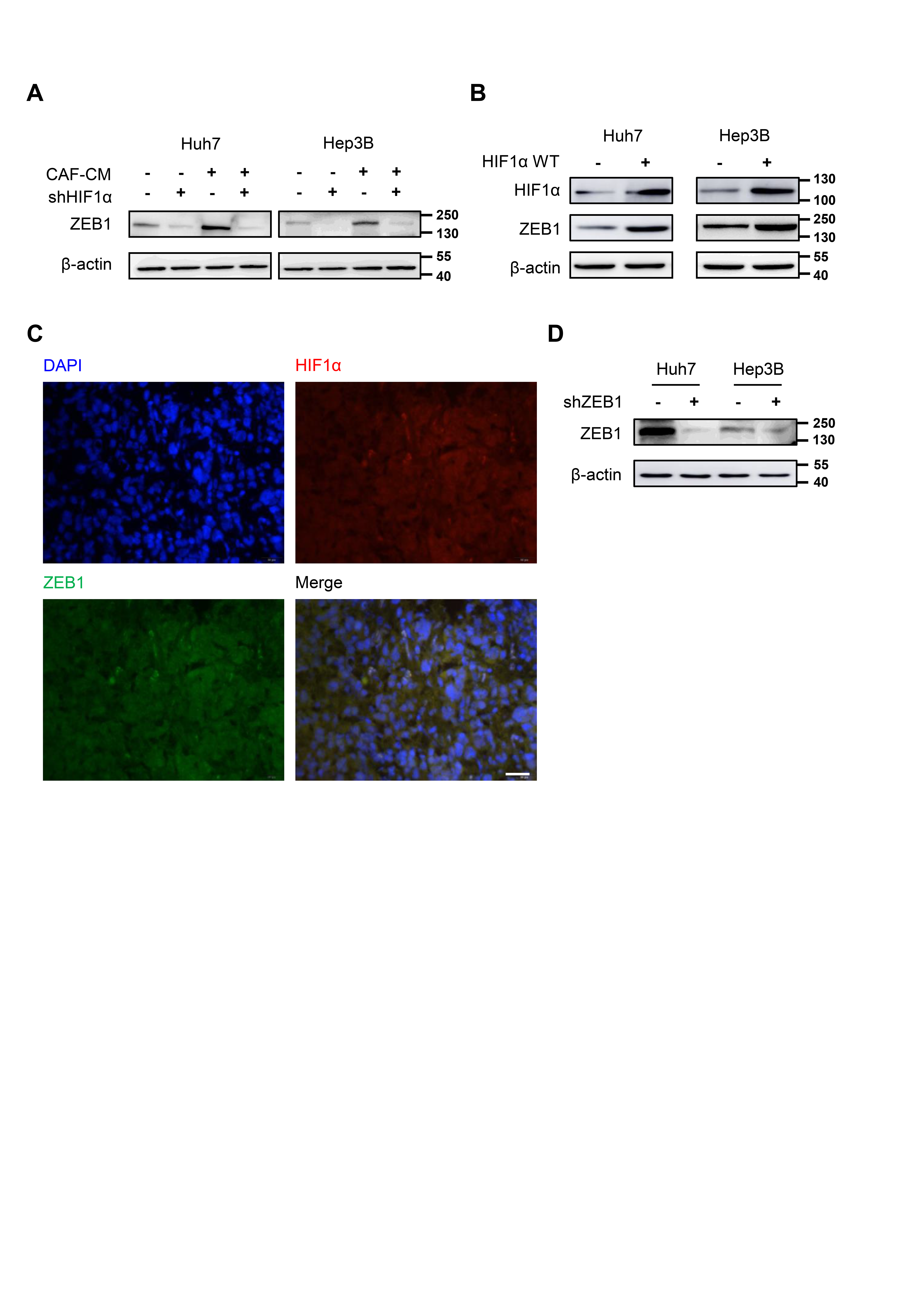

Supplement: Supplementary file 10 — Fig. S7 [file 41419_2022_4935_MOESM10_ESM.tif]
