# Supplementary material for: Cancer associated fibroblast–derived CCL5 promotes hepatocellular carcinoma metastasis through activating HIF1α/ZEB1 axis
Source: Cell Death Dis. 2022 May 20;13(5):478. doi: 10.1038/s41419-022-04935-1 (PMC9119971; doi:10.1038/s41419-022-04935-1)
Supplement: Supplementary file 12 — Data Availability Statement [file 41419_2022_4935_MOESM12_ESM.docx]

Data Availability Statement

All data generated or analyzed during this study are included in this published article and its supplementary information files. Data sharing is not applicable to this article as no datasets were generated or analyzed during the current study. The datasets generated and analyzed during the current study are not publicly available due to unpublished but are available from the corresponding author on reasonable request.
