# Supplementary material for: Cancer associated fibroblast–derived CCL5 promotes hepatocellular carcinoma metastasis through activating HIF1α/ZEB1 axis
Source: Cell Death Dis. 2022 May 20;13(5):478. doi: 10.1038/s41419-022-04935-1 (PMC9119971; doi:10.1038/s41419-022-04935-1)
Supplement: Supplementary file 13 — The human medical ethics review sheet [file 41419_2022_4935_MOESM13_ESM.pdf]

天津醫科大學
TIANJIN MEDICAL UNIVERSITY

# 人的医学伦理审查表

# The human medical ethics review sheet

申请日期：2020 年 3 月 12 日 研究号：

Application Date: 12 Mar.2020 Study number：

<table>
<tr><td>项目名称(Title of project)：肝癌肿瘤相关成纤维细胞通过分泌 CCL5 诱导 HIF1α 表达促进肝癌转移的机制研究</td></tr>
<tr><td>项目负责人(Person in charge)：徐海旭 职称（Professional title）：讲师<br>单位（Institute）：天津医科大学</td></tr>
<tr><td>项目联系人(Contacts)：徐海旭 电话（Tel）：13652171057 信箱（E-mail）：xhx198983@sina.com</td></tr>
<tr><td>研究者(Researcher)：徐海旭</td></tr>
<tr><td>请求审查类型：☑申请项目 Application project □批准后项目 After the approval of the project<br>□延续项目 The extension project □委托项目 Entrusted project</td></tr>
<tr><td>研究项目来源（Project sources）：国家自然科学基金青年科学基金</td></tr>
<tr><td>递交审查资料（Submit a review of information）<br>☑实验方案 Experimental scheme □知情同意书 Informed consent □其他资料 Other information<br>包括：试验用品安全性资料、生产企业资质证明、试验用品提供者的资质证明。</td></tr>
<tr><td>涉及人的生物医学研究内容及研究方案摘要（The abstract of the research involving human ethics review）：<br>本项目研究肝癌肿瘤相关成纤维细胞通过分泌 CCL5 诱导 HIF1α 表达促进肝癌转移的机制，收集大约 50 例肝癌患者肿瘤/癌旁组织石蜡切片和冰冻切片进行 HE 染色、免疫组化、免疫荧光染色等处理。HE 染色观察间质细胞在肿瘤组织中的分布情况；肿瘤间质的主要成分是纤维母细胞，α-平滑肌肌动蛋白（α-SMA）是肌纤维母细胞表型的主要标志蛋白，将肝癌患者肿瘤组织和癌旁组织石蜡切片进行 α-SMA 免疫组织化学染色，观察 α-SMA 蛋白在肿瘤组织和癌旁组织的表达情况；将肿瘤组织冰冻切片进行 HIF1α 和 ZEB1 抗体双荧光染色，检测肿瘤组织中阳性 HIF1A 蛋白和阳性 ZEB1 蛋白的表达以及二者在细胞核、细胞浆的分布定位；免疫组化检测 ZEB1 在肝癌患者肿瘤组织的表达水平，分析 ZEB1 表达水平与患者基本信息和临床病理参数的相关性。<br>本课题所有样本的收集、实验都将严格遵照国际和国家伦理委员会的要求执行。</td></tr>
<tr><td>申报单位意见(Opinion of the unit):<br><br>主管领导签字(signature of the officer-in-charge)： 单位章(official seal)：<br>年(year) 月(month) 日(day)</td></tr>
<tr><td>伦理委员会审查意见 Opinion of the Ethics Committees:<br>经审查“肝癌肿瘤相关成纤维细胞通过分泌 CCL5 诱导 HIF1α 表达促进肝癌转移的机制研究”项目，将肝癌患者肿瘤/癌旁组织石蜡切片和冰冻切片进行 HE 染色、免疫组化、免疫荧光染色，将在征得受试者知情同意后进行，经校伦理委员会审核，此项目符合卫生部《涉及人的生物医学研究伦理审查办法（试行）》及赫尔辛基宣言关于生物学人体试验的相关规定，同意开展研究。<br><br>天津医科大学伦理委员会<br>the Ethics Committees of Tianjin Medical University</td></tr>
</table>
